# Act on Numbers: Numerical Magnitude Influences Selection and Kinematics of Finger Movement

**DOI:** 10.3389/fpsyg.2017.01481

**Published:** 2017-08-30

**Authors:** Rosa Rugani, Sonia Betti, Francesco Ceccarini, Luisa Sartori

**Affiliations:** Department of General Psychology, University of Padova Padova, Italy

**Keywords:** kinematics, action planning, action execution, mental number line, SNARC effect

## Abstract

In the past decade hand kinematics has been reliably adopted for investigating cognitive processes and disentangling debated topics. One of the most controversial issues in numerical cognition literature regards the origin – cultural vs. genetically driven – of the mental number line (MNL), oriented from left (small numbers) to right (large numbers). To date, the majority of studies have investigated this effect by means of response times, whereas studies considering more culturally unbiased measures such as kinematic parameters are rare. Here, we present a new paradigm that combines a “free response” task with the kinematic analysis of movement. Participants were seated in front of two little soccer goals placed on a table, one on the left and one on the right side. They were presented with left- or right-directed arrows and they were instructed to kick a small ball with their right index toward the goal indicated by the arrow. In a few test trials participants were presented also with a small (2) or a large (8) number, and they were allowed to choose the kicking direction. Participants performed more left responses with the small number and more right responses with the large number. The whole kicking movement was segmented in two temporal phases in order to make a hand kinematics’ fine-grained analysis. The Kick Preparation and Kick Finalization phases were selected on the basis of peak trajectory deviation from the virtual midline between the two goals. Results show an effect of both small and large numbers on action execution timing. Participants were faster to finalize the action when responding to small numbers toward the left and to large number toward the right. Here, we provide the first experimental demonstration which highlights how numerical processing affects action execution in a new and not-overlearned context. The employment of this innovative and unbiased paradigm will permit to disentangle the role of nature and culture in shaping the direction of MNL and the role of finger in the acquisition of numerical skills. Last but not least, similar paradigms will allow to determine how cognition can influence action execution.

## Introduction

Humans usually represent numbers on a mental number line (MNL), oriented from left-to-right. Along the MNL, small numbers are placed on the left side and large numbers on the right side of space (Dehaene, 2011). The seminal experimental demonstration of the left-to-right oriented MNL, which has been extensively replicated over time, is the SNARC effect (spatial-numerical association of response codes; Dehaene et al., 1993). This effect shows that adult humans are faster in responding to small numbers on the left side of space, and in responding to large numbers on the right side of space. More recently, it has been assumed the existence of multiple spatial mappings which comprises an association between number and vertical space, as well as an association between number and near/far space (Winter et al., 2015).

Up to now, a large body of literature has replicated the evidence that number processing can modulate response times, but few studies investigated whether number processing could affect the selection of the responses. The first attempt to study this facet of the SNARC was conducted by Daar and Pratt (2008), using a free-response task. They presented participants with a stimulus, which could be a number (either a small or a large one) or a neutral character, on the central part of a monitor. Participant were required to press either a left or a right button on a keyboard (free choice task) as soon as the stimulus turned from white to green. When participants responded to small numbers they performed more left-key presses. Similarly, in responding to large numbers, they produced more right key-presses. This shows that numerical magnitude not only affects the response’s speed (as previously demonstrated in other researches) but also the direction of the choice. This evidence suggests that the spatial representation of numerical magnitude could influence which of two responses is selected for action (Daar and Pratt, 2008). The “free response” task used by Daar and Pratt (2008) differs from the “forced-choice” tasks previously and largely used to study spatial-numerical association. In these kind of tasks, indeed, participants were forced to emit a lateral response, usually to press a left- or a right-side key. In a classical “forced-choice” task, a group of participants are for example required to press a key on the left to indicate whether a number is even and a right key when it is odd, while complementary instructions are given to a second group of participants. Interestingly, even if participants are not required to estimate numerical magnitude, responses to small numbers are faster on the left side and responses to large numbers are faster on the right side of the space: SNARC effect (Dehaene, 2011). Conversely, a “free response” task allows to investigate what response is spontaneously selected. Moreover, adopting kinematic measures instead of response times provides a more fine-tuned analysis of movement, a larger range of degrees of freedom and a more sensitive investigation. In fact, a growing number of studies are now using motion capture and detailed kinematic analyses to parameterize behavior and to deeply examine questions relating to cognitive processing in naturalistic protocols (for reviews, see Castiello, 2005; Krishnan-Barman et al., 2017). From this fascinating perspective, an essential improvement of the actual knowledge would be obtained by combining a “free response” task with a kinematic analysis of movement, which may allow to understand how the responses are executed (Rugani and Sartori, 2016). In fact, given that cognitive representations of perceptual and semantic information cannot be fully understood without considering their impact on actions (Gallese and Lakoff, 2005), the existing knowledge on MNL will be advantaged by studies that analyze the motor action while responding to a number.

Semantic information related to magnitude influences indeed movement kinematics, as shown by a few studies. In a reach-to-grasp study, participants were instructed to grasp one of two identical objects, which differed solely by a word (i.e., “large” or “small”) labeled on them. Grip aperture varied accordingly with the dimension indicated by the word: it was larger for the large-labeled object and smaller for the small-labeled object (Gentilucci et al., 2000, but see also Glover and Dixon, 2002; Glover et al., 2004). Up to now, only a couple of studies has investigated the functional connection between numerical cognition and action planning (for a review see Gianelli and Fischer, 2016). Lindemann et al. (2007) asked participants to emit an odd/even judgment on Arabic digits, by grasping either a small or a large object, which required respectively a precision or a power grip. In response to small numbers precision grip movements began faster, and power grips began faster in response to large numbers. The impact of numerical magnitude on both response times and grip kinematics suggests that representations of number and representations of action share codes within a common magnitude representation’s system (Lindemann et al., 2007). In another study Gianelli et al. (2012) presented participants with a digit (from 1 to 9 without 5) and asked them to grasp a small cube and to change its position before verbally judging whether the presented number was smaller or larger than 5. Both the grip aperture and the spatial dislocation of the cube were modulated by the number magnitude, showing that the processing of magnitude is strictly related to the mechanisms underlying spatial orienting and action execution (Gianelli et al., 2012). Nevertheless, this effect, as well as the data by Daar and Pratt (2008), could also reflect a highly overlearned motor association between numerical magnitudes and manual responses, which allows to perform very efficient actions in everyday life (Schwarz and Keus, 2004). It is indeed well-known that context can influence the SNARC. When adult humans are required to relate numbers to locations on a ruler, they show a classical (left-to-right) SNARC effect. But when they are required to relate numbers on a clock face, they show an inverted (right-to left) SNARC effect (Bächtold et al., 1998). In everyday life, we often perceive and act on spatially organized numbers: rulers, keyboards, and objects ordered by their dimensions by different labels (e.g., small, medium large) or Arabic digits (1, 2, 3, 4) to indicate their sizes, are clear examples of this bias. These frequent experiences could induce us to respond to small numbers with the left hand and to larger number with the right one, as well as to prepare a smaller grasping action in relation to smaller digits (Rugani and Sartori, 2016).

Here, we aimed to investigate the association between numbers and space, combining a “free response” task with the kinematic analysis of movement, in order to understand whether number processing can influence action selection and action execution. Participants wore a miniaturized soccer shoe and kicked a small ball with their right index finger. This specific type of action has recently been adopted to elicit cognitive processing and action planning (Betti et al., 2015). In the present study, participants were instructed to kick the ball toward one of two identical little soccer goals, one located on their left and the other on their right, as soon as a stimulus was presented on a monitor screen. We designed this new and unusual task to limit the influence of learned associations between numbers and motor behavior. On 60% of the trials, the stimulus consisted of a left- or right-directed arrow and participants were instructed to kick the ball toward the direction indicated by the arrow, as fast as they could. On 40% of the trials – intermixed with arrows presentation – was instead presented a numerical stimulus that could be a small (2) or a large (8) numerical stimulus. In order to compare the effect of symbolic (i.e., abstract representations of numerical magnitudes; Vogel et al., 2015) and non-symbolic numbers (i.e., when the numerical magnitude can be extrapolated from an array of elements; Feigenson et al., 2004), in 20% of the trials participants were presented with numerals (2 or 8 digit) and the remaining 20% of the trials participants were presented with arrays composed of 2 or 8 dots. In both cases participants were free to choose toward which direction to kick the ball. The first aim of the study is to investigate what action will be selected by participants in response to a numerical value. Based on previous literature (Daar and Pratt, 2008), we expect that a small number will bias the response toward the left (i.e., the ball will be kicked more often toward the left soccer door than toward the right one) and that a large number will bias the response toward the right (i.e., the ball will be kicked more often toward the right soccer door than toward the left one). The second aim of the study is to investigate the functional connection between numerical cognition and action planning. Based on previous findings by Lindemann et al. (2007), we predict that a small number will facilitate the responses on the left side and *vice versa* that large numbers will facilitate responses on the right side (e.g., reduced action duration before contact on the target).

## Materials and Methods

### Participants

Nineteen students (13 males and 6 females, mean age = 22.89 years, *SD* = 2.38) took part in the experiment. All participants were right handed, had normal or corrected-to-normal vision, and were naive about the purpose of the experiment. Participants gave their written consent before the experiment. The experimental procedures were approved by the Ethics Committee of the University of Padova and were carried out in accordance with the principles of the 1964 Declaration of Helsinki.

### Experimental Stimuli and Stimuli Presentation

**FIGURE 1:**
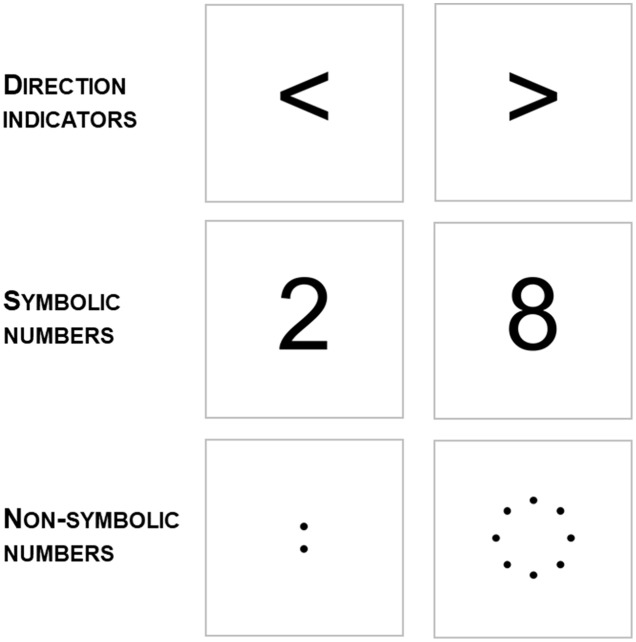
Three stimulus types were adopted in the Experiment. Direction indicators (i.e., a left and a right arrow); symbolic numbers (i.e., a small and a large digit); non-symbolic numbers (i.e., an array composed of two elements and an array of eight elements).

Stimuli consisted in: (i) direction indicators: a left arrow (<) and a right arrow (>); (ii) symbolic numbers: a small digit (2, hereafter labeled as S2) and a large digit (8, hereafter labeled as S8); (iii) non-symbolic numbers: an array composed of two elements (hereafter labeled as NS2) and an array composed of eight elements (hereafter labeled as NS8; see **Figure 1**). For the non-symbolic numbers, the elements consisted in black dots of 1 cm in diameter. In the two-element array, the dots were vertically aligned and separated by a distance of 2 cm. In the eight-element array the dots were arranged in circle (circle’s diameter was of 10.5 cm and elements were located 3 cm away one another). Both dispositions of the elements in the arrays were selected in order to avoid any explicit indication of direction. Arrows and digits were in Arial font, black color and 160 size. On each trial, a black fixation cross (7.5 cm by 7.5 cm, in Arial font, black color) appeared. After a 1000 ms delay, the fixation cross was replaced with a single stimulus.

### Apparatus and Experimental Procedure

**FIGURE 2:**
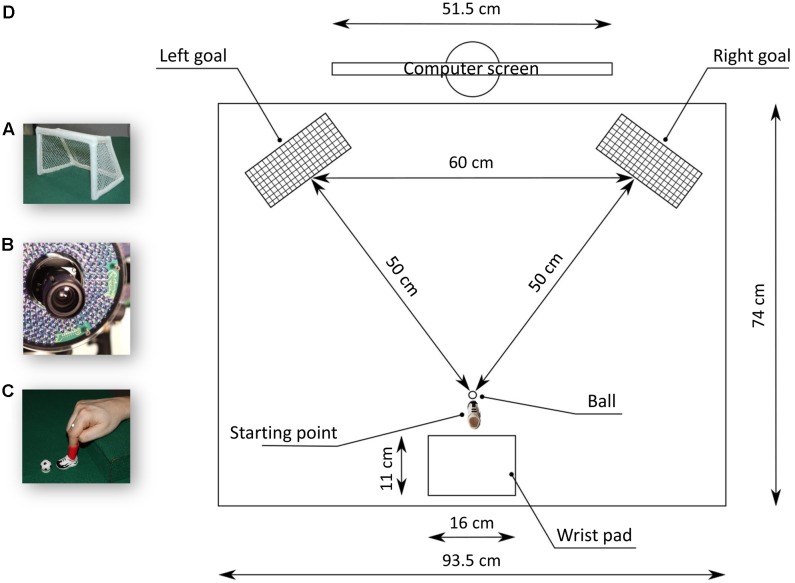
Set up and stimuli. Two soccer doors **(A)** were disposed laterally with respect to the participant’s position. A 3D-Optoelectronic SMART-D system was used to track the kinematics of the participant’s right upper limb by means of six video cameras **(B)**. An infrared reflective markers was taped to the participant’s index finger **(C)**. Participants sat in front of a monitor wearing a small soccer shoe positioned on a footprint, in front of a plastic ball **(D)**.

Participants sat on a chair in front of a table (90 cm × 90 cm) with the left wrist resting on their left leg and the right hand located in the designated start position. The experimental apparatus consisted in a work plan (93.5 cm × 74 cm) covered by a green velvet cloth. Participants’ right index was introduced in a plastic sock (4.5 cm high, 2.5 cm internal diameter) of a small plastic soccer shoe (the dimensions of the sole of the shoe were: 3 cm long, 1.5 cm wide), for a schematic representation of the apparatus see **Figure 2D**. At the beginning of each trial, participants were instructed to position the sole of the shoe on a light blue footprint (3 cm long, 1.5 cm wide), depicted on the velvet cloth, located on the midline. A plastic ball (2.3 cm of diameter) was positioned on a circle plastic support (diameter of 1.5 cm) located at 0.2 cm away from the footprint. In the start position, participants were required to rest their right wrist on a support (a pillow which was 16 cm long, 11 cm wide and 6.5 cm high), which was shaped to guarantee a comfortable and repeatable posture of the right participants’ hand, allowing them to equally and easily kick the ball either toward the left or the right.

Two small soccer goals (18 cm long 16 cm high; see **Figure 2A**) were located 50 cm away from the footprint, separated from each other by a distance of 60 cm and rotated by 30° with regard to the horizontal axis (see **Figure 2D**). A 24″ monitor (resolution 1920 × 1080 pixels, refresh frequency 120 Hz) set at eye level (the eye-screen distance was 80 cm) was used to present the experimental stimuli. Participants underwent two experimental sessions (i.e., Training and Testing) and were instructed to kick the ball toward a soccer goal as soon as a stimulus appeared, at their own pace. During training trials (*N* = 20) participants kicked the ball in the direction indicated by an arrow (*N* = 10 pointing leftward and *N* = 10 rightward, presented in random order). The aim of this session was to get the participants acquainted in kicking the ball in both directions. During test trials (*N* = 100) participants were required to kick toward the direction indicated by the arrow in the 60% of the trials. They were instead free to choose the kicking direction upon presentation of symbolic and non-symbolic numbers only in the 40% of the trials in order to maintain the free-response a sporadic event and to avoid the adoption of fixed response strategies.

Arrows, digits and array of elements were intermixed in a semi-random order (i.e., the same stimulus could not appear in more than two consecutive trials). Left and right arrows were presented in 30 trials each and both symbolic and non-symbolic numbers were presented in 10 trials each.

### Kinematics Recording

A 3D-Optoelectronic SMART-D system (Bioengineering Technology and Systems, B|T|S|) was used to track the kinematics of the participant’s right index. One light-weight infrared reflective marker (0.25 mm in diameter; B|T|S|) was taped on the index finger’s proximal phalange to measure the kicking component of the action (see **Figure 2C**). A second marker was located on the midline between the two little soccer goals, at a distance of 30 cm from each of them, and at a distance of 40 cm from the footprint. This second marker allowed to compute the finger’s location in relation to the midline. Six infrared video cameras (sampling rate 140 Hz), detecting the markers’ positions in a 3D space, were placed in a semicircle at a distance of 1–1.2 m from the table, see **Figure 2B**. Each camera position, roll angle, zoom, focus, threshold and brightness were calibrated and adjusted to optimize marker tracking before each experimental session. Static and dynamic calibrations were then carried out. For the static calibration, a three-axes frame of five markers at known distances from each other was placed in the middle of the table. For the dynamic calibration, a three-marker wand was moved throughout the workspace of interest for 60 s. The spatial resolution of the recording system was 0.3 mm over the field of view. The standard deviation of the reconstruction error was 0.2 mm for the x, y, and z axes.

### Data Processing

As concerns behavioral data, the number of right and left kicks was calculated, separately for stimulus type and magnitude (S2, S8, NS2, and NS8). Following kinematic data collection, each trial was individually checked for correct marker identification and the SMART-D Tracker software package (B|T|S|) was used to provide a 3-D reconstruction of the marker positions as a function of time. The data were then filtered using a finite impulse response linear filter (transition band = 1 Hz, sharpening variable = 2, cut-off frequency = 10 Hz; D’Amico and Ferrigno, 1990, 1992). The measurements were made along the three Cartesian axes [i.e., X (left–right), Y (up–down), and Z (anterior–posterior)]. Movement onset was defined as the time at which the tangential velocity of the finger marker crossed a threshold (5 mm/s) and remained above it for longer than 500 ms. End of movement was defined as the time at which the finger reached the maximum extension on the Y axis, after the ball was kicked. In order to specifically investigate the temporal aspects of the movement with a fine-grained analysis, we divided the whole kicking movement in two phases: Kick Preparation and Kick Finalization, computed with respect to maximum trajectory deviation. The following temporal kinematic parameters were extracted for each individual movement using a custom Protocol run in Matlab, 2014b (The 4 Math Works, Natick, MA, United States):

*Movement Time (MT)*: the time interval between movement onset and end of movement (ms);
*Time of Maximum Left Deviation (TMLD)*: the time at which the index trajectory was at a maximum distance toward the left from the midline (i.e., an imaginary line connecting the footprint and the central marker) (ms);
*Time of Maximum Right Deviation (TMRD)*: the time at which the index trajectory was at a maximum distance toward the right from the midline (ms);
*Kick Preparation (KP):* the time interval between movement onset and maximum trajectory deviation (ms);
*Kick Finalization (KF):* the time interval between maximum trajectory deviation and the end of movement (ms).

In addition, each kinematic parameter was normalized with respect to movement time, so that individual differences were accounted for.

*% Time of Maximum Left Deviation (%TMLD)*: the percentage of time at which the index trajectory was at maximum distance toward the left from the midline (%);
*% Time of Maximum Right Deviation (%TMRD)*: the percentage of time at which the index trajectory was at maximum distance toward the right from the midline (%).
*% Kick Preparation (%KP)*: the time interval between movement onset and maximum trajectory deviation (ms);
*% Kick Finalization (%KF)*: the time interval between maximum trajectory deviation and the end of movement (ms).

For each participant and kinematic index, Mean and SD was calculated, separately for each stimulus type (S2, S8, NS2, NS8, <, >) and kicking direction (left, right). The first 10 trials for each arrow direction (left or right) constituted the neutral baseline given that participants neither had to process the numerical information nor decide the direction in which kicking the ball. Baseline was calculated in this way because, while responding to arrows, participants did not have to process the numerical information and they did not have to decide the direction in which kicking the ball, since it was explicitly indicated by the arrow.

### Data Analysis

#### Behavioral Analysis

For each participant and for each trial, the means (+ SD) percentages of left kicks were computed as: (number of left choices/10) × 100. By using this formula, values of around 50% indicated no preference for kicking toward either direction; values > 50% indicated a preference for kicking toward the left; and < 50% indicated a preference for kicking toward the right. A repeated-measures ANOVA on Stimulus type (symbolic or non-symbolic) and Magnitude (small vs. large numbers) was computed on the percentage of left kicks. Significant departures from chance level (50%) were estimated by one-sample two-tailed *t*-tests.

#### Kinematic Analysis

The mean value for each parameter of interest and for each participant was compared with the corresponding neutral baseline (i.e., kicks performed in response to the first 10 arrows indicating the same direction). For example, kicks in which participants kicked toward the left in response to a symbolic stimulus were compared with the first 10 test trials in which participants responded to left arrows. Then, for each type of stimulus we compared, using a *t*-test, the means for each index with the relative neutral baseline. Bonferroni’s correction for multiple comparisons was adopted to prevent Type-1 errors. Crucially, data concerning the two different movements (i.e., left and right kicks) were considered separately and compared to their respective baseline due to mechanical and anatomical differences.

## Results and Discussion

### Behavioral Results

The repeated-measures ANOVA on left kicks showed a significant main effect of Stimulus type [*F*_(1,18)_ = 8.16, *p* < 0.05, $\eta_{p}^{2}$ = 0.31] and Magnitude [*F*_(1,18)_ = 5.89, *p* < 0.05, $\eta_{p}^{2}$ = 0.25]. Symbolic stimuli elicited more left kicks than non-symbolic stimuli. In terms of magnitude, participants chose more frequently to kick toward the left soccer door in response to a small number presentation rather than in response to a large number presentation. In particular, one-sample *t*-tests against 50% chance value revealed that, responding to a small number (S2, NS2), participants kicked the ball toward the left statistically more often than chance level [*t*_(18)_ = 3.01; *p* < 0.01]. On the contrary, in response to a large number (S8, NS8), participants kicked the ball toward the left at chance level [*t*_(18)_ = 1.01; *p* = 0.32]. Specifically, participants kicked the ball toward the left statistically more often than chance level [*t*_(18)_ = 3.73; *p* < 0.01] for S2, but not for S8 [*t*_(18)_ = 1.56; *p* = 0.13], NS2 [*t*_(18)_ = 1.73; *p* = 0.10], or NS8 [*t*_(18)_ = 0.18; *p* = 0.86].

### Kinematic Results

The fine-grained analysis of temporal phases revealed distinct patterns of movement for small and large symbolic numbers.

#### Symbolic Stimuli

**FIGURE 3:**
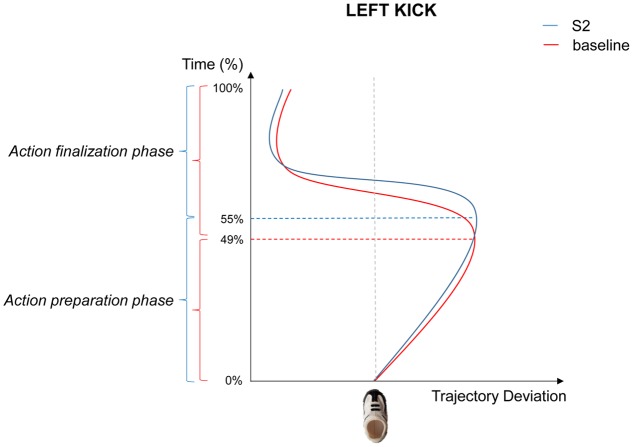
Graphical representation of the mean trajectories for left kicks following S2 presentation (blue), and left arrows (red). During the Kick Preparation phase, trajectories are directed to the opposite side with respect to the kick direction (i.e., rightward) and show different temporal patterns across conditions.

As concerns left kicks in response to small number (S2), MT was significantly longer with respect to baseline values [437.532 vs. 418.246, respectively; *t*_(18)_ = 2.606, *p* = 0.018]. In particular, a statistically significant delay of TMRD during the Preparation Phase was noticed compared to the baseline in both absolute [235.405 vs. 197.556 ms, respectively; *t*_(18)_ = 3.685, *p* = 0.002] and relative [55 vs. 49%, respectively; *t*_(18)_ = 2.302, *p* = 0.034; see **Figure 3**] terms. A longer Preparation Phase for left kicks in response to a small number implies that a shorter Finalization Phase was then performed compared to the baseline [45 vs. 51%, respectively; *t*_(18)_ = -2.302, *p* = 0.034]. No statistically significant differences were noticed for left kicks in response to a large number (S8; all *p*_s_ > 0.05).

**FIGURE 4:**
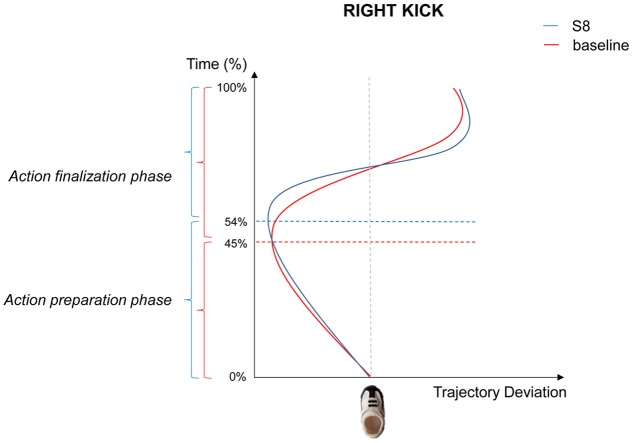
Graphical representation of the mean trajectories for right kicks following S8 presentation (blue), and right arrows (red). During the Kick Preparation phase, trajectories are directed to the opposite side with respect to the kick direction (i.e., leftward) and show different temporal patterns across conditions.

As concerns right kicks in response to large number (S8), the experimental manipulation did not affect MT compared to the baseline (*p* = 0.232), suggesting that either anatomical constraints limited the degrees of freedom during action execution, or that compensative strategies were adopted in order to maintain a constant movement duration (i.e., the Isochrony Principle; Sartori et al., 2013). However, a statistically significant delay of TMLD during the Preparation Phase was noticed compared to the baseline in both absolute [215.599 vs. 171.416 ms, respectively; *t*_(18)_ = 3.119, *p* = 0.006] and relative [54 vs. 45%, respectively; *t*_(18)_ = 2.860, *p* = 0.015; see **Figure 4**] terms. A longer Preparation Phase for right kicks in response to a large number implies that a shorter Finalization Phase was then performed compared to the baseline in both absolute [189.188 vs. 217.814 ms, respectively; *t*_(18)_ = -2.608, *p* = 0.018] and relative [46 vs. 55%, respectively; *t*_(18)_ = -2.680, *p* = 0.015] terms. No statistically significant differences were noticed for right kicks in response to a small number (S2; all *p*_s_ > 0.05).

#### Non-symbolic Stimuli

No statistically significant differences were noticed for NS2 (all *p*_s_ > 0.05) and NS8 (all *p*_s_ > 0.05), suggesting that non-symbolic stimuli did not affect kinematics.

## Conclusion

This study focused on the investigation of the MNL from an innovative perspective. To date, the majority of studies have investigated the association between numbers and space by analyzing response times, whereas researches focused on response selection, and also on more subtle measures, such as kinematic indexes, are rare. The aim of the present research was to fill this gap in the scientific literature. We designed a new, unusual and therefore not overlearned paradigm that combines a “free response” task with a hand kinematic analysis of movement. This allowed to understand *what* responses will be selected and *how* such responses are executed. Participants seated in front of a monitor and two laterally placed little soccer goals. They were required to kick, with their right index, a small ball toward either soccer goal as soon as they were presented with a stimulus. Three types of stimuli have been used: arrows (left or right) which explicitly indicated the kicking direction; symbolic numbers (digits 2 and 8) and non-symbolic numbers (array composed of 2 or 8 elements). Both types of numerical stimuli did not explicitly indicate a direction, unless the numerical magnitude could influence the chosen direction. Behavioral results showed that a small symbolic number prompted participants to selectively produce more kicks directed toward the left, while this bias did not emerge in responding to non-symbolic numbers. This suggests that the spatial representation of numerical magnitude plays a role in determining which of two responses was selected for action.

More interestingly, numerical magnitude affected the execution of a same action. We analyzed the kinematic parameters of the hand action, by dividing the whole kicking movement in two parts: Kick Preparation and Kick Finalization. Kinematics analysis revealed an effect of both small and large numbers on the timing for action execution. In responding to small numbers toward the left and to large number toward the right, participants took longer in preparing the action but they were faster to finalize the action. Crucially, the same kinematic parameter (i.e., the time of maximum trajectory deviation) was influenced in a complementary fashion for small and large symbolic numbers. S2 specifically altered the temporal aspects of left kick whereas S8 specifically modified right kicks.

Our evidence is in line with previous scientific literature. In kinematic terms, we adopted a spatial trajectory measure (i.e., trajectory deviation) that has been proved to be sensitive to participant’s motor intentions (Georgiou et al., 2007; Becchio et al., 2008), revealing that the motor system incorporates overarching goals into the action plan. Previous studies demonstrated that the trajectory path was increased and that the deviation anticipated for highly demanding actions with respect to simpler actions (Becchio et al., 2008; Sartori et al., 2009). Here, we found that our experimental manipulation highly influenced this measure leading to anticipated time intervals between maximum trajectory deviation and the end of movement. This seems to suggest that the processing of numerical magnitude increases executive load during Kick Preparation but then facilitates Kick Finalization. It could be argued that the sample size adopted in this study was too small. However, previous influential literature adopting the same kinematic approach recruited a similar number of participants. We are therefore quite confident that the sample size was appropriate for this methodological approach (see for example Grosjean et al., 2009; Hardwick and Edwards, 2012; Ménoret et al., 2013).

For what concerns action selection, a previous study, based on the presentation of non-lateralized stimuli but on the emission of lateralized responses, have found similar results. Daar and Pratt (2008), using a free-response task, reported that participants produced more left key-presses in responding to small numbers and more right key-presses in responding to large numbers. The relation between numbers and space has also been studied using other kinds of innovative paradigms. The magnitude of numbers randomly generated by adults humans was influenced by the side (left or right) they were facing. When participants turned their head toward the left they produced smaller numbers than when their head was turned toward the right (Loetscher et al., 2008). The influence of numerical magnitude on action has been demonstrated also during walking. Participants were required to generate random numbers while walking and to make a lateral turn. When the last numbers generated were relatively small participants turned left, while when the generated numbers were relatively large they turned right. Interestingly enough, lateral turn decisions could be predicted by the last few numbers generated prior to turning, suggesting an influence between numerical cognition and action (Shaki and Fischer, 2014). Also eyes movements are affected by numerical magnitude. Adults presented with a small digit (1 or 2) shifted their attention toward the left, while when presented with a large digit (8 or 9) they shifted their attention toward the right. This indicates that merely looking at numbers produces a corresponding shift of attention in the visual field (Fischer et al., 2003). More recently, by using a Posner-like task and non-symbolic numerousness (e.g., an array of dots), the effect of numerical magnitude on eye movements has been documented also in 8–9 month-old infants. Infants oriented their visual attention toward the left peripheral region of space in response to small numbers, while they oriented attention toward the right in response to large numbers (Bulf et al., 2015). These results suggest that the association between numbers and space occurs before the writing and reading acquisition undermining the idea that SNA is exclusively determined by culture. Data in support of this are obtained using a manual bisection paradigm. de Hevia and Spelke (2009) tested spatial-numerical association in adults, school children and pre-school children. All participants were required to indicate the midpoint of lines flanked by arrays composed of a different number of dots; the dots themselves essentially were an ‘irrelevant’ information. Participants of all ages presented the same bias: they bisected the line toward the right when the larger number of dots was shown in that direction (for similar results, see also Stöttinger et al., 2012). This phenomenon has been interpreted as a sort of ‘cognitive’ illusion by which the side ipsilateral to the larger (or smaller) numerosity is represented as longer (or shorter) and therefore the bisection bias toward the larger number compensates for this illusion. Data on the non-cultural origin of the spatial-numerical association are also supported by evidence on non-human animals. Day-old domestic chicks were trained to circumnavigate a panel located in the center of the apparatus and depicting a certain number of elements. At test they were presented with two panels, one located on the left and one on the right. When the panels depicted a number of elements smaller than the one experienced during training, birds circumnavigated the left panel. When the panels depicted a larger number, chicks circumnavigated the right panel (Rugani et al., 2015). Overall these findings showed that numerical magnitude influenced what was the selected response, suggesting that the coded magnitude information may reflect a link between numerical processing and actions (Rugani and Sartori, 2016). An effect of numerical magnitude on action selection in month-old and even day-old infants and in almost naïve animals suggest that SNA could be independent from everyday experience (for a review and discussion on this topic see McCrink and Opfer, 2014; Rugani and de Hevia, 2016). Our current results support this idea. Crucially, in our experiment we adopted a response task unbiased by intrinsic references to spatial-numerical representations (as you have using keyboards, for example) and we noticed that numerical magnitude influences action selection and execution. This indicates that the responses of our participants were spatially biased by the numerical magnitude of the digit, also when performing a very unusual action. For what concerns the connection between numerical cognition and action planning, results of the few studies conducted up to now found comparable results. When participants were required to respond to numbers by grasping a small or a large object, they initiated faster a precision grip when responding to small numbers and a power grip when responding to large numbers (Lindemann et al., 2007). Similarly, participants required to grasp a wooden block and to move it according to the parity status of the numeral depicted on the block showed a larger grip aperture in grasping blocks depicting larger numbers than in grasping blocks of identical size but depicting small numbers (Andres et al., 2008b). In a subsequent study, participants were required to respond to the color of the ink with which digits were written on identical objects. Numerical magnitude, even if was task-irrelevant, affected grip aperture (Namdar et al., 2014). It has also been demonstrated that numerical magnitude processing influences the free choice of an object position (Gianelli et al., 2012). Participants were required to grasp a cube and to change its position, while performing a numerical discrimination task (i.e., indicating whether a presented digit was smaller or larger than 5). When responding to small numbers compared to larger ones, participants positioned the cube more leftward and closer to themselves. Moreover, in the initial phase of the grasp movement the grip aperture was modulated by the numerical magnitude (Gianelli et al., 2012).

While the association of left and right respectively to small and large numbers in previous literature could be explained by highly overlearned motor associations between numerical magnitudes and manual responses (Schwarz and Keus, 2004), our new and unusual task suffers less this objection. Moreover, our task enables the study of the association between actions and numerical magnitudes by means of kinematic analysis of movement. In future studies it will be interesting to selectively investigate the effect of numerical magnitude on the kinematic parameters of a same identical movement (e.g., kicking the ball always toward a central goal). This would avoid the left bias that we overall noted in the present task, due to the degrees of freedom of the right finger in relation with the anatomy of the right hand. The index is in fact asymmetrically more limited in its range of action by the middle finger on its right than by the thumb on its left.

Recent accounts have underlined the importance of finger-counting in number processing, as it leaves its mark in adulthood (Di Luca et al., 2006; Fischer, 2008), and it helps developing associations between numbers and hand actions. The origin of the relationship between numerical skills and finger counting is supported by different research. Abacus experts, while solving arithmetic calculation, show spontaneous hand movements (Hatano et al., 1977). Hubbard et al. (2005) suggested that the relation between finger counting and numerical cognition (the manumerical cognition hypothesis) could also explain why finger agnosia, left–right confusion and dyscalculia often co-occur in the Gerstmann syndrome.

The deep relation between numerical cognition and action has been explicated on the embodied cognition theory. This assumes that activation of bodily representations can help the comprehension of abstract concepts (Glenberg, 1997). As for other fields of cognition (Fischer and Zwaan, 2008) the embodied cognition theory has been proposed also for numerical concepts (Andres et al., 2008a; Lindemann et al., 2009). A challenge that this perspective offers concerns the use of embodied numerical cognition and associated movement tasks in teaching numerical concepts (Goldin-Meadow et al., 2009; Moeller et al., 2012). Moving hands help children in solving numerical problems (Moeller et al., 2012). Interestingly, enough, the positive effect of movement on numerical problem solving it is not limited to finger and/or hands but it is extended to the whole body. Different groups of first-graders participated to different trainings. A full-bodily experience training required to show the position of numbers by walking on a number line depicted on the floor. A non-full-bodily-experience training required to indicate the location of number on a tablet screen, using a computer-mouse. The full-bodily-experience training affected more positively the performance on math tasks than those who participated to a number line training which did not required a full-bodily experience (Link et al., 2013).

However, this is the first experimental demonstration of the relation between number cognition and motor action in a new and not-overlearned context. The employment of this paradigm will permit to untangle, from a very innovative perspective, the influence of biological and cultural factors in shaping the direction of the MNL and the role of finger in the acquisition of numerical skills. Moreover, our paradigm could be easily used to test whether the association between motor responses and space can be obtained also with auditory stimuli (for a recent demonstration of a spatial numerical association with auditory numerical stimuli, see Klichowskia and Króliczak, 2017). Last but not least, similar paradigms will allow to determine how cognition can influence action execution. A very recent paper by Pinheiro-Chagas et al. (2017) has in fact investigated the relation between simple arithmetic calculation (single-digit additions and subtractions) and finger movements. Participants were asked to point to the result of an arithmetic computation on a number line, while finger trajectory was constantly monitored. The analysis of trajectories unveiled that, during calculation, the two operands were serially processed. The finger first pointed toward the larger operand, then slowly deviated toward the correct result. This slow deviation was showed in subtractions and additions and it was proportional to the magnitude of the smaller operand (Pinheiro-Chagas et al., 2017). This evidence supports a previous finding on simpler numerical tasks (Song and Nakayama, 2009), highlighting that even complex mental operations can be continuously reflected in finger-pointing movements (Pinheiro-Chagas et al., 2017).

The employment of our innovative paradigm will not solely allow to understand the role of culture in shaping the direction of MNL, but it will also represent a simple and powerful method to disentangle the role of fingers in the acquisition of numerical skills.

## Ethics Statement

The experimental procedures were approved by the Institutional Review Board at the University of Padua and were in accordance with the Declaration of Helsinki (Sixth revision, 2008).

## Author Contributions

RR and LS designed the study, created the stimuli, collected, analyzed the data, RR, FC, and LS did the statistical analyses; RR, SB, and LS interpreted the data discussed the results; SB, RR, and LS created the figures; RR and LS wrote the manuscript; SB and FC critically revised the manuscript.

## Conflict of Interest Statement

The authors declare that the research was conducted in the absence of any commercial or financial relationships that could be construed as a potential conflict of interest.
